# Gene expression signatures associated with sensitivity to azacitidine in myelodysplastic syndromes

**DOI:** 10.1038/s41598-020-76510-7

**Published:** 2020-11-11

**Authors:** Kyuryung Kim, Silvia Park, Hayoung Choi, Hye Joung Kim, Yong-Rim Kwon, Daeun Ryu, Myungshin Kim, Tae-Min Kim, Yoo-Jin Kim

**Affiliations:** 1 Department of Medical Informatics, College of Medicine, The Catholic University of Korea, Seoul, Korea; 2 Cancer Research Institute, College of Medicine, The Catholic University of Korea, 222 Banpo-daero, Seocho-Gu, Seoul, 06591 Republic of Korea; 3 Department of Biomedicine and Health Sciences, College of Medicine, The Catholic University of Korea, Seoul, Korea; 4 Seoul St. Mary’s Hematology Hospital, College of Medicine, The Catholic University of Korea, Seoul, Korea; 5 Catholic Genetic Laboratory Center, Department of Internal Medicine, Seoul St. Mary’s Hospital, College of Medicine, The Catholic University of Korea, 222 Banpo-daero, Seocho-Gu, Seoul, 06591 Republic of Korea; 6 Laboratory of Hematological Disease and Immunology, Convergent Research Consortium for Immunologic Disease, College of Medicine, The Catholic University of Korea, Seoul, Korea; 7 Department of Laboratory Medicine, College of Medicine, The Catholic University of Korea, Seoul, Korea; 8 Leukemia Research Institute, College of Medicine, The Catholic University of Korea, Seoul, Korea

**Keywords:** Cancer, Molecular biology, Diseases, Medical research, Oncology

## Abstract

Allogeneic stem cell transplantation is currently the only curative treatment option for myelodysplastic syndromes (MDS). Pre-transplant debulking treatment have been employed for advanced MDS and we previously reported that marrow response (blast ≤ 5%) following the bridging therapy with hypomethylating agent was an independent favorable factor for survival; however, it is still not clear which patients will respond to hypomethylating agent and which genomic features can predict the response. In this study, we performed RNAseq for 23 MDS patients among which 14 (61%) and 9 (39%) patients showed marrow complete remission and primary resistance to azacitidine, respectively. Differential expression-based analyses of treatment-naive, baseline gene expression profiles revealed that molecular functions representing mitochondria and apoptosis were up-regulated in responders. In contrast, we identified genes involved in the Wnt pathway were relatively up-regulated in non-responders. In independent validation cohorts of MDS patients, the expression of gene sets specific to non-responders and responders distinguished the patients with favorable prognosis and those responded to azacitidine highlighting the prognostic and predictive implication. In addition, a systems biology approach identified genes involved in ubiquitination, such as *UBC* and *PFDN2*, which may be key players in the regulation of differential gene expression in treatment responders and non-responders. Taken together, identifying the gene expression signature may advance our understanding of the molecular mechanisms of azacitidine and may also serve to predict patient responses to drug treatment.

## Introduction

Myelodysplastic syndromes (MDS) are clonal stem cell disorders characterized by peripheral blood cytopenia due to ineffective hematopoiesis and by their potential to evolve into acute myeloid leukemia (AML)^1^. The ineffective hematopoiesis is associated with elevated apoptosis of hematopoietic clones, a phenomenon that may be related to signaling mediated by TRAIL ligands/receptors^2^, *FAS* molecules^3^ and/or myelosuppressive *TGFβ*^4^. Excessive apoptosis occurs in the early stages of MDS and declines as MDS progresses toward the leukemic stage. About one third of MDS patients progress to AML, which is probably associated with expansion and evolution of subclones with certain mutations at the stem cell level^5,6^. The genetic profiles of individual patients are heterogeneous and an individual patient’s unique genetic makeup may impact their clinical phenotype, prognosis, and response to therapy. An MDS patient’s genome frequently harbors somatic mutations which may contribute to disease progression; however, there is no single gene that appears to be sufficient to elicit disease and the majority of genes mutated in MDS are present in fewer than 5% of cases, highlighting the complexity of the disease^7,8^. These heterogenity of MDS have led to the development of risk-based stratification systems, and treatment options such as hypomethylating agent (HMA) or allogeneic stem cell transplantation (SCT) are selected systematically according to risk groups^9–11^.

The use of HMA, such as azacitidine (AZA) and decitabine, has become a standard treatment for higher-risk MDS patients, as HMA confer a survival benefit over conventional care^12,13^, but many issues, including determining the optimum dose or duration of treatment, remain to be addressed. Importantly, it is still not clear which patients will respond to HMA or not. A number of predictive genomic markers of the response to HMA treatment have been reported among the frequent genomic aberrations in MDS genomes. For example, mutations in genes involved in DNA methylation (*DNMT3A*, *TET2*, *IDH1*, and *IDH2*) and genes encoding epigenetic regulations (*ASXL1*, *EZH2*, and *TET2)* have been proposed to predict AZA sensitivity^14–16^, but the clinical relevance of these associations remains controversial^17–19^. A DNA methylation-based scoring system using a suite of 10 genes has been proposed, but while the score was correlated with patient survival, it could not predict clinical responses to demethylating agents^20^. The expression of a number of other genes, such as *BCL2L10*^21^, *FAS*^22^, and *PI-PLCbeta1*^23^, may also be indicative of HMA activity. Moreover, associations between repressed expression of anti- *DNMT1* miRNA*s* and HMA resistance have been noted^24^. All of these findings have contributed substantially to a better understanding of individual variability in HMA responses among MDS patients; However, mutation, methylation, or expression information involving limited genes are not full enough to understand disease heterogeneity with respect to different response to HMA treatment. In this regard, genome-wide profiling of mRNA expression may facilitate comprehensive prediction of drug sensitivity.

One important usage of HMA is to serve as a bridge to SCT by diminishing marrow blasts to an acceptable level prior to transplantation^25^. Several retrospective studies have shown that pre-SCT HMA could be a feasible alternative to induction chemotherapy^26–28^ even for patients with excessive blasts or AML^29^. As the influence of HMA response on transplant outcomes is still controversial, we previously completed a retrospective analysis of 98 patients who received HMA for higher-risk MDS with > 5% marrow blasts^30^. Our study showed that continued marrow response at the time of SCT was an independent positive predictor of overall and disease-free survival after transplantation. In light of our findings and those of another study in which a positive correlation between marrow clearance and genomic mutations was reported^31^, we aimed to identify the molecular markers that could predict a patient’s marrow response to HMA.

In this study, we performed RNAseq-based gene expression profiling of advanced MDS patients with excess blasts (EB) prior to their receiving AZA treatment. Comparison of gene expression profiles between 14 responders and 9 non-responders revealed a number of molecular functions (e.g., apoptosis and cellular respiration) were relatively activated in responders. Functional scores of these molecular pathways were also correlated with patient survival in an independent MDS cohort, and the results suggested their potential prognostic value.

## Results

### Sequencing data from enrolled patients

A total of 23 patients who received AZA for MDS with excess blasts 1 (MDS-EB-1; *n* = 9) and MDS-EB-2 (*n* = 14) before SCT were enrolled in the study (Table 1). The non-responder group consisted of 9 cases showing primary resistance, which included 8 cases with disease progression to AML (*n* = 6) or MDS-EB-2 (*n* = 2) and 1 case with stable disease without any hematological improvement (SD-HI) after 5 cycles of AZA treatment. The responder group consisted of cases with complete remission (CR) (*n* = 7) or marrow CR (mCR) with or without any hematological improvement (*n* = 7). The patients’ bone marrow derived mononuclear cells were subjected to transcriptome sequencing by RNAseq. The sequencing-related information is presented in Supplementary Table 1.

**Table 1:** Clinicopathological features of the study patients.

No.	Sex/age	Characteristics at treatment			Mutations*	Azacytidine treatment and outcomes	
		WHO	BM blasts	Cytogenetic profile		Response (cycles)	Course and survival (months)
1	M/63	MDS-EB-2	10%	46,XY [20]	*LAMB4*	CR (3)	Died of post-SCT AML (19.6)
2	M/68	MDS-EB-2	19%	46,XY,t(1;3)(p36.1;q21) [18]/46,XY [2]	*SF3B1*	CR (9)	Died of secondary resistance and progression to AML (12.0)
3	F/55	MDS-EB-1	8%	46,XX,t(7;11)(p15;p15) [20]	NA	CR (4)	Died of post-SCT AML (24.2)
4	M/65	MDS-EB-1	8%	46,XY,del(5)(q22),del(9)(q13),del(11)(q23),add(18)(q23) [6]/46,XY [3]	NA	CR (5)	Died of post-SCT relapse (29.1)
5	F/59	MDS-EB-2	15%	46,XX [20]	NA	CR (5)	Alive in post-SCT relapse (40.1)
6	F/71	MDS-EB-1	7%	46,XX [20]	NA	CR (6)	Alive in secondary resistance (46.3)
7	M/71	MDS-EB-2	10%	46,XY [20]	NA	CR (4)	Alive in post-SCT remission (30.3)
8	M/66	MDS-EB-2	15%	47,XY, + 8 [20]	*NRAS, TET2, ETV6, KRAS*	mCR + HI-E/P (6)	Died of secondary resistance (17.2)
9	M/55	MDS-EB-2	11%	46,XY [20]	NA	mCR + HI-E (8)	Alive in post-SCT remission (32.6)
10	M/75	MDS-EB-2	8%	46,XY [20]	NA	mCR-HI-E (6)	Died of secondary resistance and progression to AML (21.8)
11	M/53	MDS-EB-1	8%	46,XY, + 1,der(1;15)(q10;q10) [20]	*JAK2, ETV6*	mCR-HI (2)	Died of post-SCT toxicity (6.2)
12	F/41	MDS-EB-1	8%	45,-X,t(X;9;14)(q25;q34;q11.2) [20]	Not detected	mCR-HI (2)	Alive in post-SCT remission (71.5)
13	F/68	MDS-EB-2	15%	46,XX,del(20)(q11.2) [20]	NA	mCR-HI (2)	Died of brain hemorrhage (9.2)
14	M/59	MDS-EB-2	17%	46,XY [20]	*TET2, ASXL1, RUNX1*	mCR-HI (5)	Died of post-SCT toxicity (13.5)
15	M/77	MDS-EB-1	6%	47,XY, + 8 [20]	NA	1° DP to AML (1)	Died of AML (1.1)
16	M/73	MDS-EB-2	12%	46,XY [20]	NA	1° DP to AML (2)	Died of AML (11.4)
17	M/67	MDS-EB-1	9%	46,XY,t(11;19)(q23;p13.1) [14]/45,idem,dic(11;17)(p15;p13) [1]/46,XY [5]	NA	1° DP to AML (6)	Died of AML (7.6)
18	F/38	MDS-EB-2	10%	46,XX,del(20)(q11.2q13.1) [7]/46,idem,del(12)(p11.2p12) [9]/46,XX [4]	*EZH2*	1° DP to AML (2)	Died of AML (2.7)
19	M/42	MDS-EB-2	10%	46,XY [20]	NA	1° DP to AML (4)	Alive in post-SCT remission (40.7)
20	M/52	MDS-EB-2	17%	46,XY [20]	*NRAS, EZH2*	1° DP to AML (3	Died of post-SCT relapse (21.4)
21	M/71	MDS-EB-1	6%	46,XY [20]	NA	1°DP to EB-2 (2)	Alive in AML (43.5)
22	M/62	MDS-EB-1	8%	45,X,-Y [2]/46,XY [18]	NA	1°DP to EB-2 (6)	Alive in post-SCT remission (42.3)
23	M/64	MDS-EB-2	19%	46,XY [20]	NA	SD-HI (5)	Died of post-SCT AML (24.6)

Note that mutations are present for 8 cases available and NA represents that sequencing and other mutation-related information are not available.

*BM bone marrow, DP* disease progression, *CR* complete remission, *mCR* marrow complete remission, *SD* stable disease, *HI* hematologic improvement, *E* erythrocyte, *P* platelet, *SD-HI* stable disease without any hematologic improvement, *SCT* stem cell transplantation, *MDS-EB* myelodysplastic syndrome with excess blasts, *AML* acute myeloid leukemia.

*Eight out of 23 patients were analyzed using targeted deep sequencing as previously demonstrated^19^. A total of 26 well-known genes in MDS (*DNMT3A, TET2, EZH2, RUNX1, ASXL1, STAG2, CBL, TP53, SRSF2, SF3B1, U2AF1, LAMB4, DNMT1, ETV6, KRAS, NF1, NPM1, NRAS, PRPF8, IDH1, IDH2, JAK2, FLT3, SETBP1, ATRX, and ZRSR2*) were included in a customized panel.

### Differential-expression-based identification of marker genes and molecular functions

**Figure 1:**
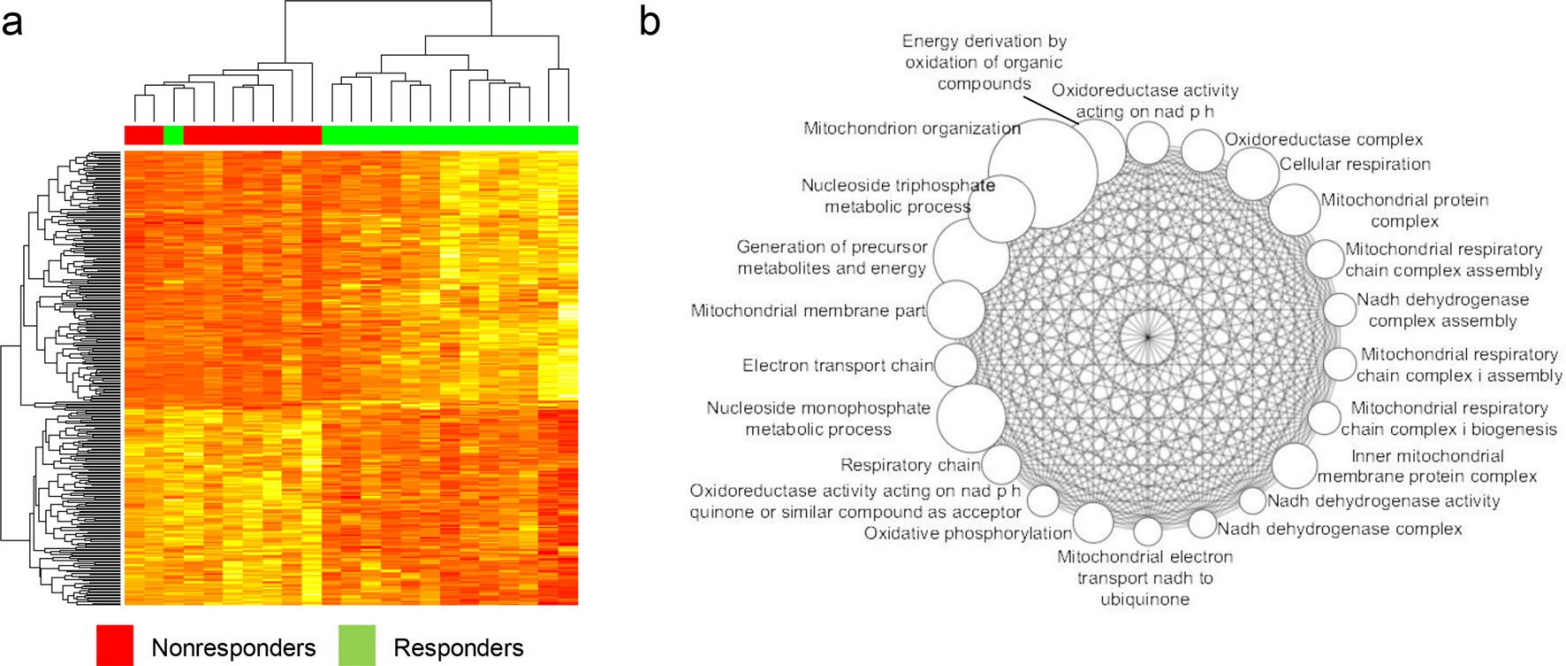
Hierarchical clustering and functional enrichment analysis of DEGs. (**a**) The 300 DEGs (*P* < 0.01; *t*-test, unadjusted) are shown in a heatmap with gene- and sample-wise dendrograms by hierarchical clustering. Non-responders (red; *n* = 9) are largely segregated from responders (green; *n* = 14) except for an outlier. (**b**) Twenty-two GO categories with significant enrichment (Bonferroni corrected *P* < 0.05; Fisher's exact test) with DEGs are shown as nodes in a network. All the pairs of nodes showed significant overlap of DEGs and were connected by edges in the network. The GO annotations of 22 nodes converge on the function of ‘cellular respiration' and 'mitochondria'. The node size corresponds to the gene number of the corresponding GO categories.

We first selected differentially expressed genes (DEGs) between responders and non-responders (*n* = 300; *P* < 0.01, *t*-test, unadjusted, FPKM and Combat adjusted data). The hierarchical clustering of 300 DEGs was able to segregate responders and non-responders, with the exception of one outlier (Fig. 1a). These findings suggest the baseline mRNA expression of MDS patients may have the potential to predict sensitivity to AZA. The list of DEGs is presented in Supplementary Table 2. To identify the molecular functions associated with the DEGs, we performed Fisher's exact test with Gene Ontology (GO) categories (MSigDB C5 category) and found significant associations in 22 GO categories (Bonferroni corrected *P* < 0.05). To summarize the identified GO categories by taking into account the redundancy in GO categories, we measured the extent of overlap for all possible pairs of the 22 GO categories identified above. Of note, all possible pairs of the 22 GO categories showed significant overlap between DEGs (*P* values of Fisher's exact test in the range of 1.2E−25 to 5.4E−55), which suggested the gene–gene co-expression networks were highly interconnected with respect to GO functional categories. The 22 GO categories are illustrated in a network diagram (Fig. 1b). The examination of 22 GO functional annotations in MDS patients indicated that the DEGs between AZA responders and non-responders largely represented 'cellular respiration' and 'mitochondria'. The most significant pairing between GO categories was for the 'electron transport chain' and 'respiratory chain' (*P* = 5.4E−55; Fisher's exact test) with overlapping DEGs for *NDUFS7, COX8A, UBA52, UQCR11, NDUFA2, NDUFB7, NDUFB8, NDUFA7, NDUFB2, COX4I1, NDUFA13, ETFB, NDUFA1, NDUFB9, NDUFA11*, and *UBC*. Oxidative stress and mitochondrial dysfunction have been postulated to play a role in the development of MDS^32^. Although the association between sensitivity to AZA and mitochondrial dysfunction has not been clearly defined, a number of reports suggest that increased oxidative phosphorylation relative to glycolysis may indicate metabolic plasticity of tumor cells and is associated with the resistance to chemotherapy in colorectal cancers^33^ and targeted therapeutics in melanomas^34^.

**Table 2:** GSEA results of molecular functions enriched in AZA responders and non-responders.

Up-regulated	Molecular functions	Genes	ES*	NES*	*P* values	FDR*	FWER*
Responders	GO_RESPONSE_TO_OXYGEN_RADICAL	15	0.68	1.89	0	1	0.498
	GO_LEUKOCYTE_APOPTOTIC_PROCESS	22	0.62	1.83	0.002	1	0.756
	GO_OXIDOREDUCTASE_ACTIVITY_ACTING_ON_A_HEME_GROUP_OF_DONORS	22	0.73	1.78	0.023	1	0.841
	GO_OXIDOREDUCTASE_ACTIVITY_ACTING_ON_NAD_P_H	85	0.6	1.78	0.028	1	0.845
	GO_OXIDATIVE_PHOSPHORYLATION	82	0.74	1.78	0.02	1	0.845
	GO_REGULATION_OF_TRANSCRIPTION_ELONGATION_FROM_RNA_POLYMERASE_II_PROMOTER	23	0.67	1.78	0	1	0.852
	GO_CYTOCHROME_COMPLEX	17	0.8	1.78	0.006	1	0.855
	GO_OXIDOREDUCTASE_ACTIVITY_ACTING_ON_NAD_P_H_QUINONE_OR_SIMILAR_COMPOUND_AS_ACCEPTOR	50	0.75	1.77	0.018	1	0.875
	GO_ELECTRON_TRANSPORT_CHAIN	92	0.68	1.77	0.024	0.93	0.877
	GO_NUCLEOSIDE_MONOPHOSPHATE_METABOLIC_PROCESS	222	0.54	1.76	0.035	0.93	0.894
Non-responders	GO_EXPLORATION_BEHAVIOR	15	− 0.67	− 1.87	0	1	0.609
	GO_REGULATION_OF_MEMBRANE_DEPOLARIZATION	33	− 0.51	− 1.74	0.007	1	0.924
	GO_DENDRITE_DEVELOPMENT	70	− 0.49	− 1.74	0.01	1	0.928
	GO_REGULATION_OF_SYNAPTIC_TRANSMISSION_GABAERGIC	21	− 0.58	− 1.73	0.012	1	0.935
	GO_WNT_PROTEIN_BINDING	27	− 0.59	− 1.73	0.008	1	0.94
	GO_IONOTROPIC_GLUTAMATE_RECEPTOR_BINDING	21	− 0.53	− 1.71	0.013	1	0.962
	GO_PHOTORECEPTOR_OUTER_SEGMENT	47	− 0.44	− 1.7	0.002	1	0.962
	GO_DENDRITE_MORPHOGENESIS	36	− 0.53	− 1.69	0.013	1	0.969
	GO_REGULATION_OF_STEM_CELL_POPULATION_MAINTENANCE	15	− 0.61	− 1.68	0.014	1	0.97
	GO_BRAIN_MORPHOGENESIS	25	− 0.59	− 1.67	0.008	1	0.976

*ES, enrichment score; NES, normalized enrichment score; FDR, false discovery rate; FWER, family-wise error rate.

### Enrichment analyses

**Figure 2:**
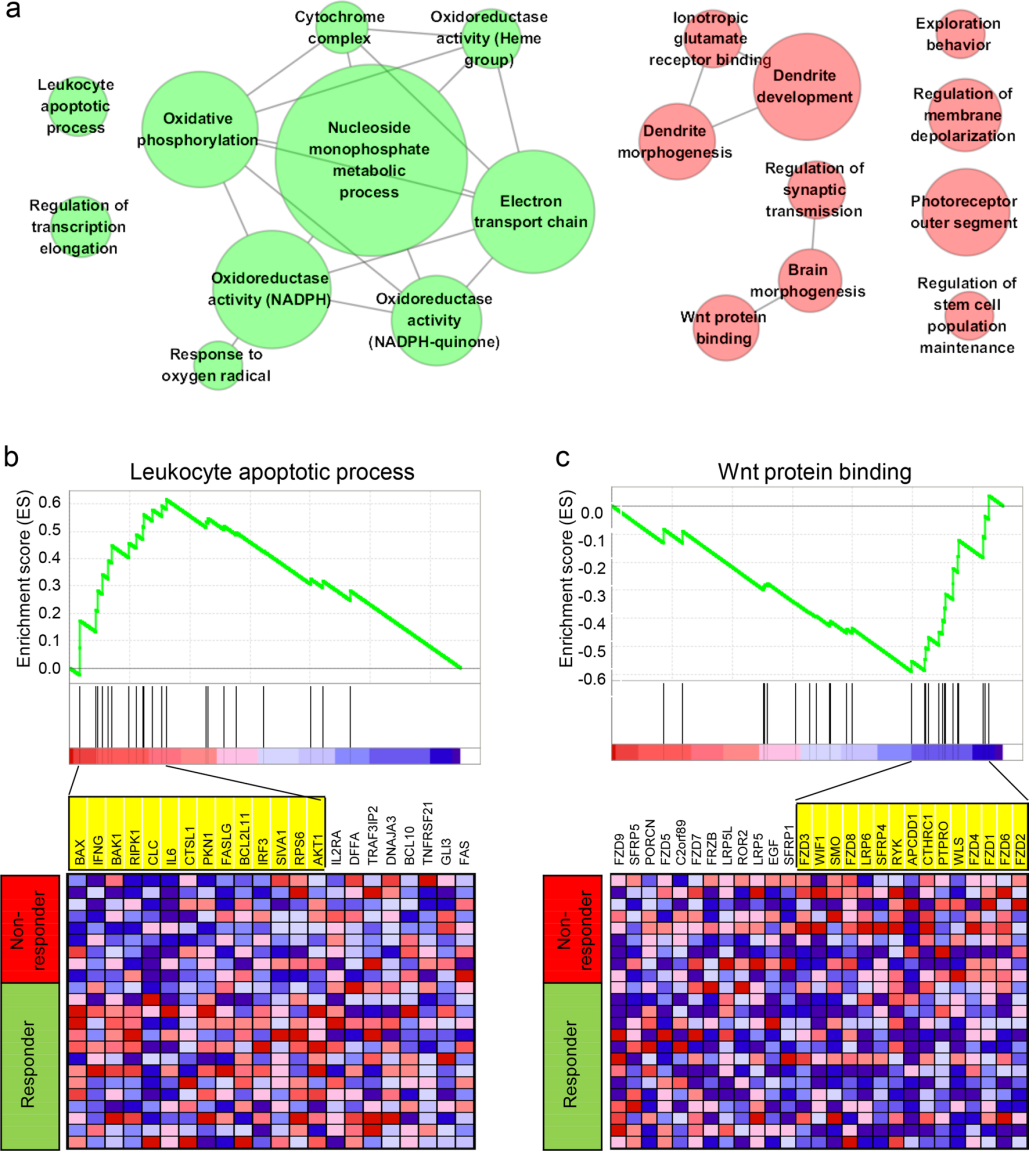
GSEA analysis. (**a**) The top 20 significant gene sets relatively up- and down-regulated (green and red, *n* = 10 and 10, respectively) in responders compared to non-responders are shown as nodes in a network. The node size is proportional to the number of genes in the gene set. Edges in the network represent the significant overlap of the leading edge genes between two nodes. (**b**) An enrichment plot of the leukocyte apoptotic process is shown as a snapshot of the GSEA analysis. A heatmap showing the expression of genes belonging to the set is shown below with the annotation of genes ordered. Yellow indicates the leading-edge gene subsets. (**c**) A similar plot of the gene set of Wnt protein binding.

To further identify subtle but functionally coordinated mRNA expression changes that cannot be captured by DEG analyses, we performed additional gene set enrichment analyses (GSEA) to identify molecular functions for which the gene members are differentially expressed between AZA responders and non-responders. Table 2 lists the 10 most significant GO categories relatively up-regulated in responders compared to non-responders, as well as 10 GO categories up-regulated in non-responders compared to responders. To summarize the GO categories according to the overlap of gene members, we also calculated the significance of the overlap of gene members and a total of 20 GO categories are shown in a network diagram (Fig. 2a). In the network, a main network composed of eight GO categories largely representing oxidative phosphorylation and the electron transport chain were found to be relatively up-regulated in responders compared to non-responders (green nodes in Fig. 2a), which is consistent with the results of the DEG analyses. In addition, GO categories of 'leukocyte apoptotic process' and 'regulation of transcription elongation' were evident, suggesting that these functions are relatively activated in responders. With respect to GO categories relatively up-regulated in non-responders, 'dendrite development/morphogenesis' (3 GO categories) and 'Wnt protein binding/brain morphogenesis' (3 GO categories) were found to be major functional categories (red nodes in Fig. 2a).

Two examples of GO categories ('leukocyte apoptosis process' and ‘Wnt protein binding’, which are up- and down-regulated, respectively, in responders) are selected to show their enrichment plots as well as their expression heat maps and leading-edge genes (Fig. 2b,c), respectively. Ineffective hematopoiesis is one of cardinal features of MDS, and is thought to be due to increased apoptosis of myeloid progenitors; the acquisition of proliferative capacity by clonal progenitors has been considered the key event in the progression of MDS to acute myeloid leukemia^8^. Thus, it is reasonable to assume that elevated expression of apoptosis-related genes at baseline may predict a favorable prognosis and responsiveness to AZA. In our study, we found no significant differences in *FAS* expression between responders and non-responders (Fig. 2b; *P* = 0.48; *t*-test, unadjusted), or between *BCL2L10* and *PLCB1* expression (*P* = 0.69 and 0.42; *t*-test, unadjusted), both of which have previously been proposed as expression markers of AZA sensitivity^21,23^. In our study, only two genes, *BAX* and *IFNG*, had significantly different expressions between the responders and non-responders (*P* = 0.03 and 0.04, respectively; *t*-test, unadjusted). Wnt/β-catenin signaling has been presumed to be involved in the ineffective hematopoiesis of patients with myeloid neoplasms with 5q deletions, and has also been presumed to have therapeutic implications in MDS through inhibition of this signaling^35^. Our results further suggest that the mRNA expression levels in Wnt/β-catenin pathway genes including a number of frizzled receptors may indicate the responsiveness to AZA with similar implications in MDS-derived stromal cells^35^. It is postulated that up-regulated expression of Wnt pathway genes at baseline in non-responders encodes cellular resistance to AZA treatment. Other GO categories that showed substantial differences in expression with respect to HMA responses are shown in Supplementary Fig. 1, in which enrichment plots and heatmaps of two metabolic functions (‘Nucleoside monophosphate metabolic process’ and ‘Oxidoreductase activity acting on NADPH’) are demonstrated.

In addition to assessing DEG and GSEA, we further employed a DeMAND network-based system biological approach^36^ to identify the genes related to the mechanism of action (MoA) of AZA. In this process, the level of perturbation was measured for individual genes and their interacting partners in regulatory networks by comparing the gene expression profiles of responders and non-responders following drug perturbation. The 20 most significant candidate MoA genes are listed in Table 3. The modeling revealed *UBC* and *PFDN2* as candidate MoA genes whose protein interactions with interacting partners were the most perturbed in the context of the regulatory network.

**Table 3:** MoA genes of AZA responder and non-responder revealed by DeMAND analysis.

Gene	*P* value (adjusted)	Fold change	Description
*UBC*	6.02E−240	− 0.39	Ubiquitin C
*PFDN2*	1.23E−94	− 0.29	Prefoldin subunit 2
*GNL3L*	2.82E−80	0.37	G protein nucleolar 3-like
*ERO1LB*	3.13E−53	0.56	Endoplasmic reticulum oxidoreductase 1 beta
*SACM1L*	7.87E−52	0.35	SAC1 suppressor of actin mutations 1-like
*PSMA2*	5.44E−46	− 0.16	Proteasome subunit alpha 2
*PSMB4*	6.62E−42	− 0.32	Proteasome subunit beta 4
*XRN1*	1.73E−41	0.62	5′-3′ exoribonuclease 1
*PSMD8*	9.68E−40	− 0.37	Proteasome 26S subunit, non-ATPase 8
*RPL26L1*	3.17E−36	− 0.18	Ribosomal protein L26 like 1
*PSMB2*	1.31E−29	− 0.13	Proteasome subunit beta 2
*NDUFAB1*	6.05E−28	− 0.31	NADH: ubiquinone oxidoreductase subunit AB1
*ZNF622*	1.42E−26	− 0.37	Zinc finger protein 622
*KRR1*	4.45E−24	0.35	Small subunit processome component homolog
*BOP1*	1.29E−23	− 0.43	Block of proliferation 1
*PSMB6*	6.53E−23	− 0.38	Proteasome subunit beta 6
*CUL2*	2.06E−22	0.28	Cullin 2
*RPLP2*	2.10E−22	− 0.43	Ribosomal protein lateral stalk subunit P2
*ERCC4*	1.29E−21	0.47	ERCC excision repair 4, endonuclease catalytic subunit
*PDIA3*	6.68E−20	− 0.19	Protein disulfide isomerase family A member 3

Positive and negative fold change values represent that the corresponding genes are up- or down-regulated in non-responders compared to responders, respectively.

### Validation of azacitidine sensitivity signatures in independent cohorts

**Figure 3:**
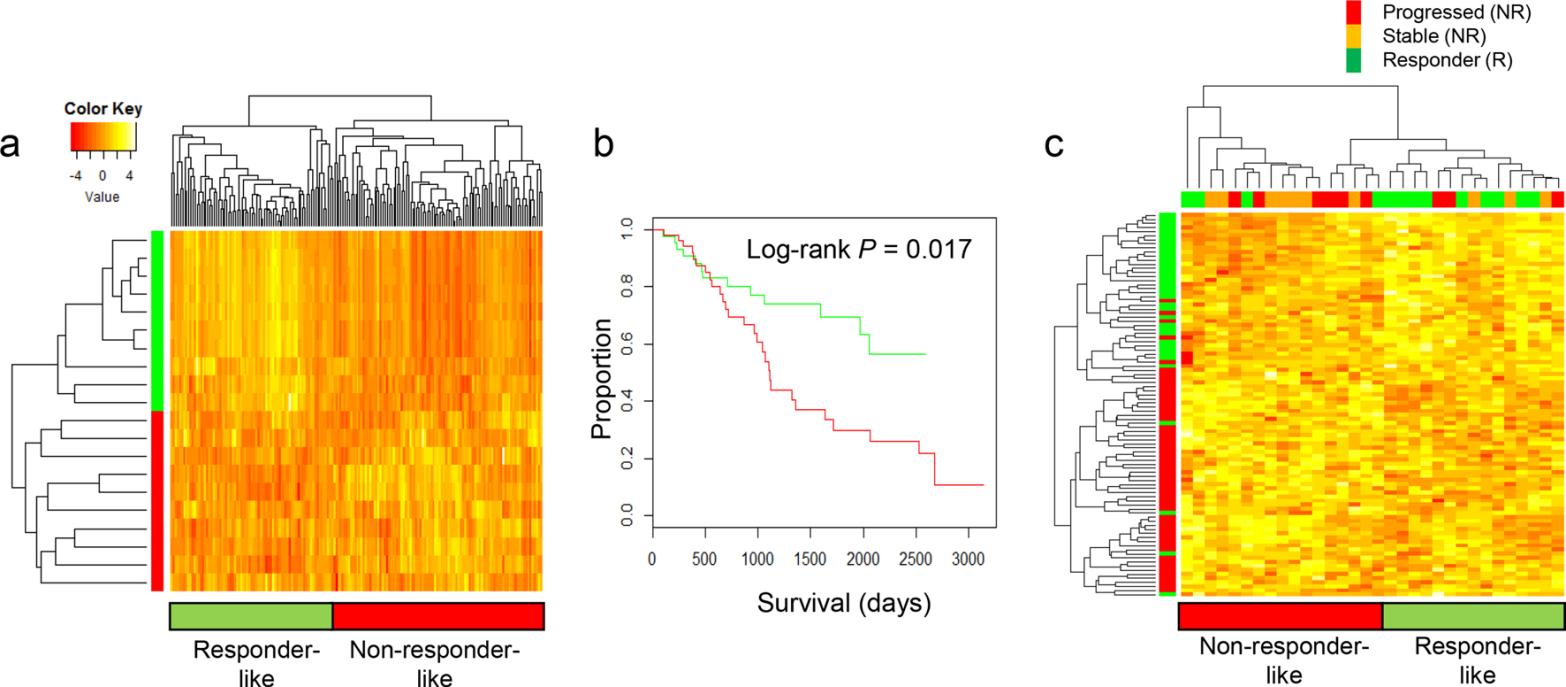
Survival analysis of independent MDS cohorts. (**a**) Clustering of marker gene expression by gene-set level scores segregated the 123 MDS patients (GSE58831) is shown with a heatmap. Column-wise green/red bars below indicate the patients whose expression profiles resemble those of azacitidine responders/non-responders, respectively (‘responder-like’ and ‘non-responder-like’) (**b**) MDS patients segregated according to expression profiles showed significant survival differences (*P* = 0.017, log-rank test). The green and red lines indicate the patients whose scores resemble those of azatidicine responders and non-responders (green and red bars below in Fig. 3a), respectively. (**c**) Clustering of marker gene expression segregated the 32 MDS patients (GSE77750) into responder-like and non-responder-like groups. Responder-like patients were enriched with AZA responders (green, top) while non-responder-like patients were enriched with non-responders (here, progressed/red and stable/orange were considered as AZA non-responders).

To confirm the impact of the identified gene markers on prognosis and HMA response, we applied the gene set-level scores (i.e., the mean expression of leading edge genes of 20 GO terms in Table 2) in independent data sets. We obtained publicly available gene expression profiles of 123 MDS patients with overall survival (GSE58831)^37^ and 32 patients with AZA treatment history (13 responders and 19 non-responders; GSE77750)^38,39^. For survival (GSE58831), MDS patients whose gene expression profiles resembled those of AZA responders in our study (‘responder-like’) showed better survival (green in Fig. 3a,b) compared to those whose gene expression resembled non-responders (‘non-responder-like’, red in Fig. 3a,b; log-rank test *P* = 0.017). This result suggests that the baseline expression profile from AZA responders may be associated with a favorable prognosis in MDS patients highlighting the prognostic implication of the identified genes in our study. For AZA responsiveness (GSE77750), the clustering using identified markers segregated the 32 MDS patients into ‘responder-like’ and ‘non-responder-like’ cases with the modest level of prediction accuracy 0.687 (Fig. 3c). Thus, our gene markers may have both prognostic and predictive implication with the AZA treatment for MDS patients.

We also tested MoA genes in an independent cohort (GSE58831) to test the prognostic values of the markers. Although not significant, the expression of MoA genes was able to segregate the MDS patients with a substantial difference in overall survival suggesting that the MoA genes, in spite of their small size, may be utilized for prognostic markers (Supplementary Fig. 2a,b).

## Discussion

Although HMA response in MDS is known to be associated with better survival both in transplant and non-transplant settings^12,30,40^, it has not been possible to clearly identify which patients will respond to drug therapy. In this study, we performed next-generation transcriptome sequencing to identify gene-expression-based predictive markers that can discriminate responders from non-responders. For prediction, we first identified a set of DEGs that can segregate the AZA responders and non-responders. The DEGs identified in this study were also able to group the patients from an independent MDS cohort into subgroups of distinguished drug response and overall survival, indicating the selected genes may potentially identify AZA responders and MDS patients with favorable or unfavorable prognosis. Given our study was based on baseline gene expression data prior to therapy, our findings may potentially support a clinical decision to select AZA as a bridging treatment for MDS patients with excess blasts.

The expression of previously proposed single genetic markers of favorable treatment outcomes, such as *FAS*, *BCL2L10,* and *PI-PLCbeta1* were not able to segregate AZA responders and non-responders in our study. One possible explanation for this discrepancy is that our study only included MDS-EB patients and the definition of a responder in this study was confined to bone marrow response and did not include either partial response or stable disease with hematological improvement. The identification of MDS at the level of single-gene markers is complicated by tumor heterogeneity: in this regard, it has been proposed that multi-gene signatures or gene-set-level classifiers may be more clinically relevant than single-gene markers. Although the repressed expression of the apoptosis-related molecule *FAS* by aberrant DNA methylation may be indicative of AZA sensitivity, the baseline expression of *FAS* was not significantly different between responders and non-responders in this study. However, we note that ‘leukocyte apoptosis process’ was identified as one of the significant GO terms showing differential expression in a set of genes in our study. Among the genes belonging to the GSEA top 20 gene set list, we identified two genes, *BAX* and *IFNG* belonging to the leukocyte apoptosis process has only significant differential expression (*P* < 0.05, t-test, unadjusted, Fig. 2b) and are up-regulated in responders compare with non-responders. The pro-apoptotic protein encoded by *BAX* is transcriptionally activated in MDS patients with more favorable survival outcomes^41^ and *IFNG* is known to trigger apoptosis in undifferentiated progenitor cells such as hematopoietic stem cells^42^. Although the potential roles of these genes in the context of marrow response to AZA require further validation in an independent cohort, the molecular pathways and marker genes identified in our functional-gene-set-level differential analyses may have clinical utility. Supplementary Fig. 3 shows the level of differential expression of *BAX* and *IFNG* were modest, e.g., ranked 429th and 442nd in the differential expression-ordered list of total genes, respectively. In addition, 'Wnt protein binding' was also identified as one of the molecular functions relatively up-regulated in non-responders at baseline. The Wnt pathway represents one of the key molecular pathways in carcinogenic processes^43^ and we assume that this pathway may provide survival signals to the tumor clones. In the survival analyses, we found that the gene set level of 20 GO terms can segregate the patients according to survival outcomes. Notably, gene expression profiles characteristic of AZA marrow responders identified in this study were significantly linked to better survival in an independent MDS cohort, which suggests that the genes identified in our study may have potential clinical utility as general prognostic markers. However, we acknowledge that the results must be interpreted with a caution given the substantial differences between our cohort and validation cohort regarding examined cells, patients’ characteristics and clinical outcomes of interests. We examined mononuclear cells in this study whereas CD34 + cells were used in two validation cohorts (GSE58831, GSE77750), although recent study by Shiozawa et al. showed the concordance of expression levels for selected genes between bone marrow CD34 + cells and mononuclear cells from MDS patients^44^. In addition, advanced MDS patients with excess blasts who were treated with HMA were only included in our study, while all subtypes of MDS patients with no information on treatment (GSE58831) or AZA treated AML patients, not only MDS patients (GSE77750) were analyzed in validation cohorts. Actually, when we selected EB patients only in validation cohort (GSE58831), the prognostic impact according to the expression of marker genes was not significant (Supplementary Fig. 2c,d), probably due to the small sample size. Based on these discrepancies between the studies along with the small sample size, our results should be explored in a further investigation.

The DeMAND algorithm was employed as a systems biology approach to identify network-level perturbation and to reveal key MoA genes. Through this process, *UBC* and *PFDN2* were designated as potential MoA proteins whose interactions were substantially perturbed by AZA treatment. Of interest, two genes have roles in protein metabolism such as ubiquitination (*UBC*) and as protein chaperone (*PFDN2*). It has been previously reported that ubiquitination is implicated in histone modification^45^ and the ubiquitin–proteasome pathway is associated with the stability of the *DNMT1* protein, findings which indicate a potential molecular link between ubiquitination and the demethylating agent AZA^46^. Therefore, we may assume that *UBC* may have a role in the regulation of gene expression associated with AZA treatment.

Taken together, the markers for the AZA sensitivity identified in this study may predict drug responsiveness prior to AZA treatment and may have general prognostic implications.

## Methods

### Patient selection

To identify molecular markers in MDS genomes associated with marrow responses following pre-transplant bridging treatment with AZA patients who received the standard schedule of AZA (75 mg/m^2^/day for 7 consecutive days) for MDS with > 5% marrow blasts and cases showing extremes of treatment response (marrow complete remission versus primary treatment failure) were screened. Response to treatment was assessed using the modified International Working Group response criteria^47^. Cases achieving CR or mCR with or without hematological improvement were categorized as responders, while non-responders consisted of those who experienced primary treatment failure defined either by primary disease progression or stable disease without hematological improvement (SD-HI)^48^. Minimum 4 cycles of AZA was administered before response assessment of SD-HI, whereas assessment of CR, mCR or disease progression was allowed to assess even before the 4th cycles. The patients did not receive previous treatment before AZA for their MDS with excess blasts. Patients with bone marrow samples available for research purposes were enrolled in the final study population. This study was approved by the Institutional Review Board of the Seoul St. Mary’s Hematology Hospital at the Catholic University of Korea, and complied with the tenets of the Declaration of Helsinki. Informed consent was obtained from all subjects.

### mRNA sequencing

Bone marrow samples (10 mL) were mixed with 0.3 mL of heparin to prevent coagulation, then diluted with 20 mL of phosphate‐buffered saline (PBS, Welgene, Daegu, Korea). The cells were then fractionated on a Lymphoprep density gradient (Axis‐Shield, Oslo, Norway) through centrifugation at 600 g for 10 min. Interface mononuclear cells were isolated and washed with PBS. An erythrocyte (RBC) lysis buffer (0.154 M NH4Cl, 10 mM KHCO3, 0.1 mM EDTA; Sigma Aldrich, St. Louis, MO) was then added to destroy contaminating RBCs. The RNA was isolated using the standard Tri-Reagent1(Sigma Chemicals) protocol. Preparation of mRNA libraries was performed using an illumina (REF. RS-122-2101 ~ 2) TruSeq stranded kit as manufacturer’s recommendation. The sequencing was performed using the NextSeq500 platform (illumina, REF. SY-415-1001). Paired-end 75 bp sequencing reads were generated and the sequencing information is presented in Supplementary Table 1. Raw sequencing reads in FASTQ files were mapped and aligned by TopHat (version.2.1.1)^49^. Transcript-level alignment was completed using Cufflinks^50^ using annotated transcripts of hg19 GTF (UCSC, TCGA.hg19.June2011.gaf). The expression of each gene was represented in terms of fragments per kilobase million (FPKM) and used for the subsequent analyses. RNA sequencing data have been submitted to the Sequence Read Archive (SRA) under accession number of PRJNA650236.

### GSEA

Gene set enrichment analysis^51^ (GSEA, version 2.0, https://software.broadinstitute.org/gsea) was used to identify differential expression of genes associated with specific molecular functions between drug responders and non-responders^52^. For molecular functions, we used Gene Ontology gene sets as available in MSigDB (MSigDB, C5:GO terms, version 6.0).

## Supplementary information

Supplementary Information.

Supplementary Figure 1.

Supplementary Figure 2.

Supplementary Figure 3.

Supplementary Table 1.

Supplementary Table 2.
