# Supplementary material for: Gene expression signatures associated with sensitivity to azacitidine in myelodysplastic syndromes
Source: Sci Rep. 2020 Nov 11;10:19555. doi: 10.1038/s41598-020-76510-7 (PMC7658235; doi:10.1038/s41598-020-76510-7)
Supplement: Supplementary file 1 — Supplementary Information. [file 41598_2020_76510_MOESM1_ESM.docx]

**Title page for the supplementary material**

**Title:** Gene expression signatures associated with sensitivity to azacitidine in myelodysplastic syndromes

**Authors’ names:**

Kyuryung Kim^1,2,3^*, Silvia Park^4,8^*****, Hayoung Choi^5^, Hye Joung Kim^6^, Yong-Rim Kwon^6^, Daeun Ryu^1,2^, Myungshin Kim^5,7^, Tae-Min Kim^1,2,3^** and Yoo-Jin Kim^4,6,8^**

**Name of institution:**

^1^ Department of Medical Informatics, College of Medicine, The Catholic University of Korea, Seoul, Korea

^2^ Cancer Research Institute, College of Medicine, The Catholic University of Korea, Seoul, Korea

^3^ Department of Biomedicine & Health Sciences, College of Medicine, The Catholic University of Korea, Seoul, Korea

^4^ Seoul St. Mary's Hematology Hospital, College of Medicine, The Catholic University of Korea, Seoul, Korea.

^5^ Catholic Genetic Laboratory Center, Seoul St. Mary’s Hospital, College of Medicine, The Catholic University of Korea, Seoul, Republic of Korea

^6^ Laboratory of Hematological Disease and Immunology, Convergent Research Consortium for Immunologic Disease, College of Medicine, The Catholic University of Korea, Seoul, Korea.

^7^ Department of Laboratory Medicine, College of Medicine, The Catholic University of Korea, Seoul, Korea.

^8^ Leukemia Research Institute, College of Medicine, The Catholic University of Korea, Seoul, Korea.

***^,^ **These authors contributed equally to this work**

**Corresponding author**

**Yoo-Jin Kim**

**Address:** Department of Internal Medicine, Seoul St. Mary's Hospital, College of Medicine, The Catholic University of Korea, 222 Banpo-daero, Seocho-Gu, Seoul, 06591, Republic of Korea

**Phone: +**82-2-2258-6057 **FAX: +**82-2-2258-2759 **E-mail:** yoojink@catholic.ac.kr

**Tae-Min Kim**

**Address:** Cancer Research Institute, College of Medicine, The Catholic University of Korea, 222 Banpo-daero, Seocho-Gu, Seoul, 06591, Republic of Korea

**Phone: +**82-2-2258-7619 **FAX: +**82-2-3477-7623 **E-mail:** tmkim@catholic.ac.kr

**Supplementary Figure Legends**

**Supplementary Figure 1. GSEA enrichment plots metabolic functions.** Among the metabolic function-related gene sets, we selected two gene sets of 'nucleoside monophosphate metabolic process' (a) and 'oxidoreductase activity acting on NADPH' (b). Half of the genes belonging to the gene sets were transcriptionally up-regulated in responders suggestive of metabolic activation of tumors that responded to AZA.

**Supplementary Figure 2.** **Prognostic impact of 20 MoA genes and 300 DEGs in validation cohort.** (a, b) 123 MDS patients of independent data set (GSE58831) are distinguished by 20 MoA genes. Below indicates the patients whose expression profiles resemble those of azacitidine responders/non-responders (responder-like and non-responder-like, respectively). (c, d) EB patients only (n=41, BM Blast make up 5% to 19%) obtained from an independent cohort (GSE58831) are analyzed by 300 DEGs, and the prognostic impact according to the expression of marker genes was not significant.

**Supplementary Figure 3.** **The level of differential expression of *BAX* and *IFNG* genes.** Genes are sorted in order of the differential expression between AZA responder and non-responder. *BAX* and *IFNG* genes are ranked 429^th^ and 442^nd^, respectively, suggesting that their level of differential expression are modest.

**Supplementary Table Legends**

**Supplementary Table 1. Sequencing related information**

**Supplementary Table 2. The list of top 300 differentially expressed genes.** Top 300 gene with variable expression were selected by fold change values. Positive and negative fold change values represent that the genes were up- and down-regulated in non-responders compared to responders, respectively. FDR is false discovery rate.
