# Supplementary material for: Gene expression signatures associated with sensitivity to azacitidine in myelodysplastic syndromes
Source: Sci Rep. 2020 Nov 11;10:19555. doi: 10.1038/s41598-020-76510-7 (PMC7658235; doi:10.1038/s41598-020-76510-7)
Supplement: Supplementary file 2 — Supplementary Figure 1. [file 41598_2020_76510_MOESM2_ESM.pdf]

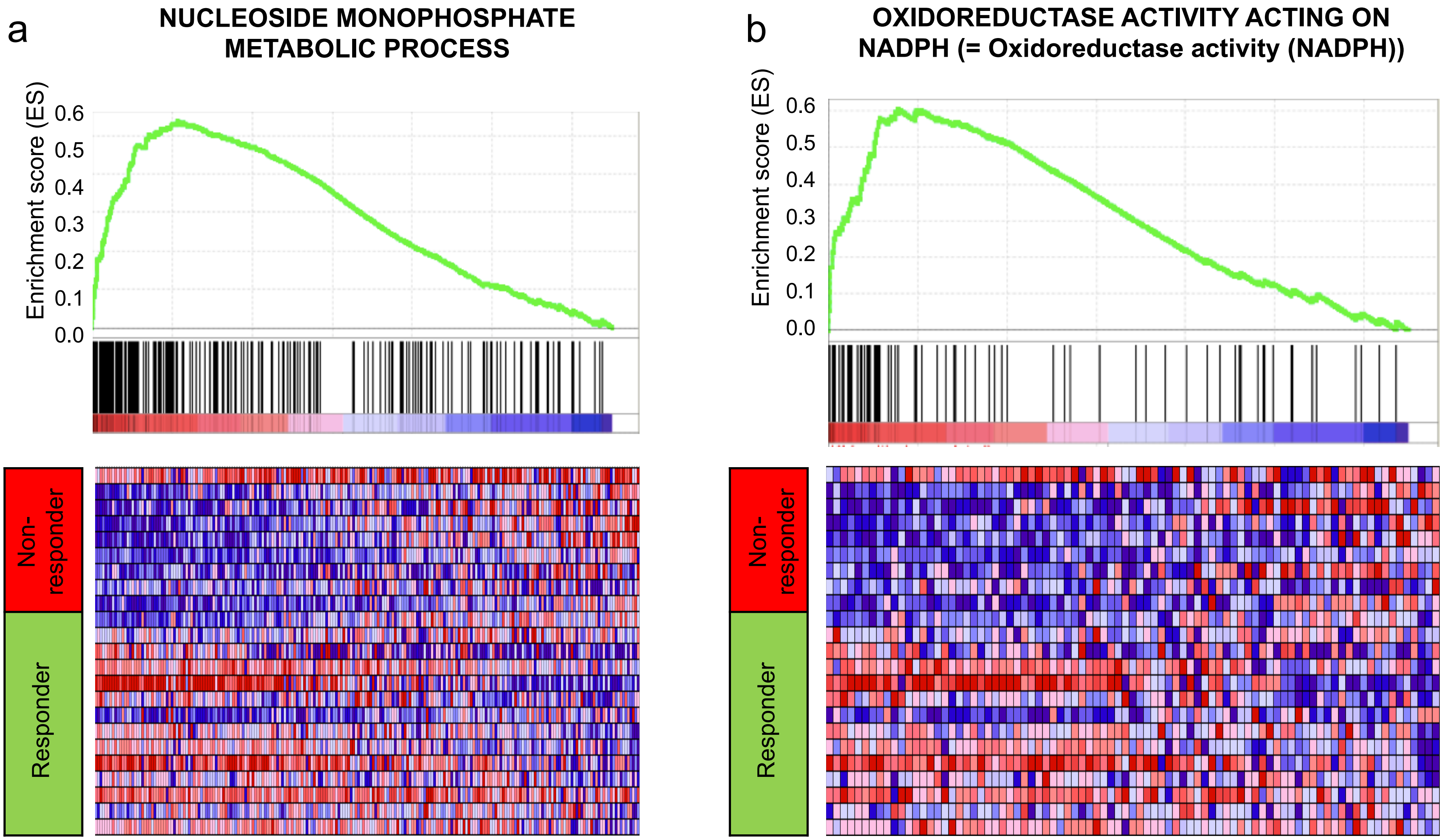

Supplementary Figure 1. GSEA enrichment plots metabolic functions. Among the metabolic function-related gene sets, we selected two gene sets of 'nucleoside monophosphate metabolic process' (a) and 'oxidoreductase activity acting on NADPH' (b). Half of the genes belonging to the gene sets were transcriptionally up-regulated in responders suggestive of metabolic activation of tumors that responded to AZA.
