# Supplementary material for: Gene expression signatures associated with sensitivity to azacitidine in myelodysplastic syndromes
Source: Sci Rep. 2020 Nov 11;10:19555. doi: 10.1038/s41598-020-76510-7 (PMC7658235; doi:10.1038/s41598-020-76510-7)
Supplement: Supplementary file 3 — Supplementary Figure 2. [file 41598_2020_76510_MOESM3_ESM.pdf]

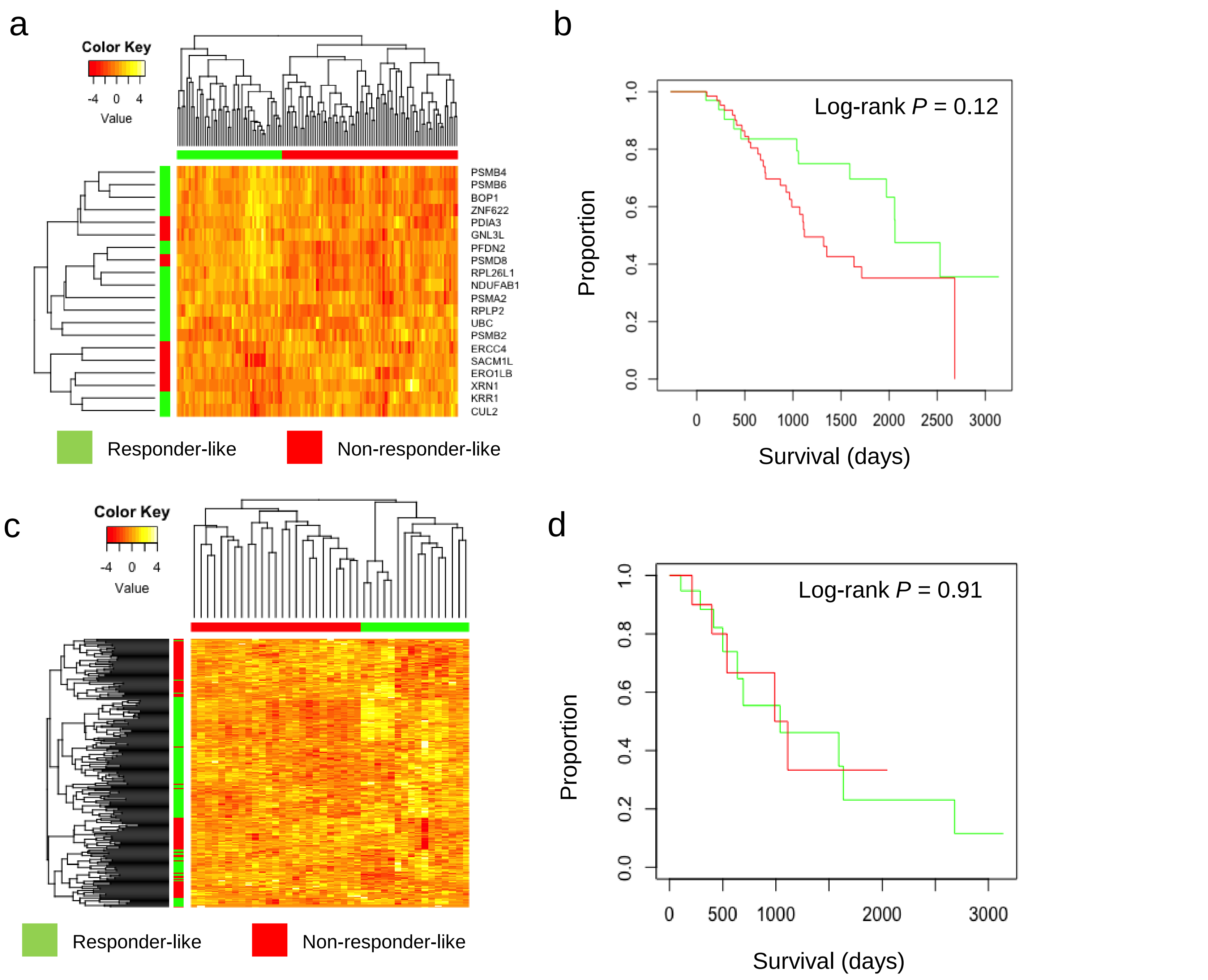

Supplementary Figure 2. Prognostic impact of 20 MoA genes and 300 DEGs in validation cohort. (a, b) 123 MDS patients of independent data set (GSE58831) are distinguished by 20 MoA genes. Below indicates the patients whose expression profiles resemble those of azacitidine responders/non-responders (responder-like and non-responder-like, respectively). (c, d) EB patients only (n=41, BM Blast make up 5% to 19%) obtained from an independent cohort (GSE58831) are analyzed by 300 DEGs, and the prognostic impact according to the expression of marker genes was not significant.
