# Supplementary material for: Gene expression signatures associated with sensitivity to azacitidine in myelodysplastic syndromes
Source: Sci Rep. 2020 Nov 11;10:19555. doi: 10.1038/s41598-020-76510-7 (PMC7658235; doi:10.1038/s41598-020-76510-7)
Supplement: Supplementary file 4 — Supplementary Figure 3. [file 41598_2020_76510_MOESM4_ESM.pdf]

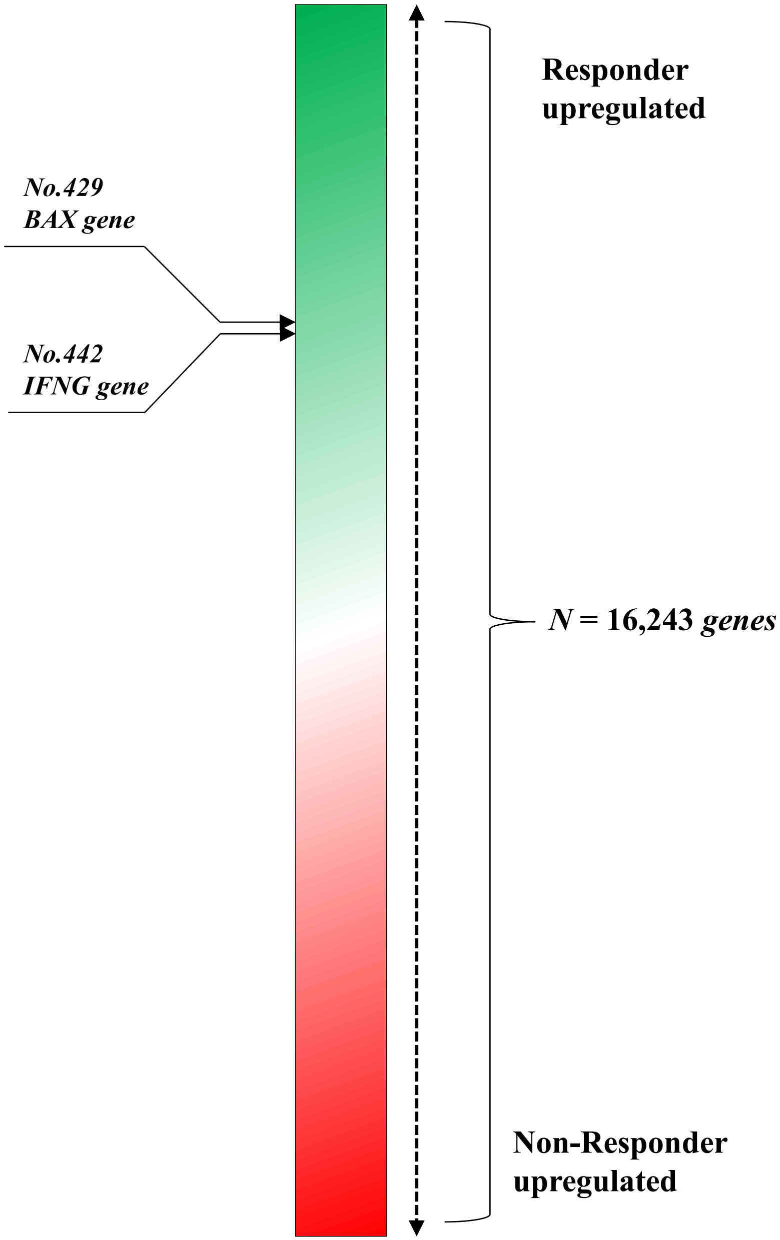

Supplementary Figure 3. The level of differential expression of *BAX* and *IFNG* genes. Genes are sorted in order of the differential expression between AZA responder and non-responder. *BAX* and *IFNG* genes are ranked 429th and 442nd, respectively, suggesting that their level of differential expression are modest.
